# Development of a time-resolved fluorescence immunoassay kit for detecting canine coronavirus and parvovirus through double labeling

**DOI:** 10.1186/s12985-024-02302-4

**Published:** 2024-03-11

**Authors:** Laiqing Li, Cuicui Chen, Huankun Liang, Wenqi Dong, V. N. Leontiev, Igor Vitalievich Voytov

**Affiliations:** 1 Belarusian State Technological University, 13a Sverdlov Str, 220006 Minsk, Minsk Belarus; 2 Guangzhou Youdi Bio-technology Co., Ltd, 510663 Guangzhou, China; 3 Guangzhou Zhenda Biopharmaceutical Technology Co., Ltd, 510663 Guangzhou, China

**Keywords:** Coronavirus, Parvovirus, Canine, Time-resolved fluorescence immunoassay, Double labeling, Kit

## Abstract

**Objective:**

Canine enteric coronavirus (CCV) and canine parvovirus type 2 (CPV-2) are the main pathogens responsible for acute gastroenteritis in dogs, and both single and mixed infections are common. This study aimed to establish a double-labeling time-resolved fluorescence immunoassay (TRFIA) to test and distinguish CCV and CPV-2 diseases.

**Methods:**

A sandwich double-labeling TRFIA method was established and optimized using europium(III) (Eu^3+^)/samarium(III) (Sm^3+^) chelates. CCV/CPV-2 antigens were first captured by the immobilized antibodies. Then, combined with Eu^3+^/Sm^3+^-labeled paired antibodies, the Eu^3+^/Sm^3+^ fluorescence values were detected after dissociation to calculate the CCV/CPV-2 ratios. The performance, clinical performance and methodology used for laboratory (sensitivity, specificity, accuracy and stability) testing were evaluated.

**Results:**

A double-label TRFIA for CCV and CPV-2 detection was optimized and established. The sensitivity of this TRFIA kit was 0.51 ng/mL for CCV and 0.80 ng/mL for CPV-2, with high specificity for CCV and CPV-2. All the accuracy data were less than 10%, and the recovery ranged from 101.21 to 110.28%. The kits can be temporarily stored for 20 days at 4 °C and can be stored for 12 months at temperatures less than − 20 °C. Based on a methodology comparison of 137 clinically suspected patients, there was no statistically significant difference between the TRFIA kit and the PCR method. Additionally, for CCV detection, the clinical sensitivity was 95.74%, and the clinical specificity was 93.33%. For CPV-2 detection, the clinical sensitivity was 92.86%, and the clinical specificity was 96.97%.

**Conclusion:**

In this study, a double-label TRFIA kit was prepared for CCV and CPV-2 detection with high laboratory sensitivity, specificity, accuracy, stability, clinical sensitivity and specificity. This kit provides a new option for screening/distinguishing between CCV and CPV-2 and may help improve strategies to prevent and control animal infectious diseases in the future.

## Introduction

Coronaviruses are enveloped RNA viruses that can cause respiratory, enteric, or systemic diseases in a variety of mammalian hosts [1]. Recent human viral pandemics, including severe acute respiratory syndrome (SARS), Middle East respiratory syndrome (MERS), and COVID-19, demonstrate the zoonotic potential of coronaviruses and could have devastating impacts [2]. Canine enteric coronavirus (CCV) is a notable coronavirus in companion animals [3]. Although CCV can initially cause only mild gastrointestinal clinical signs, several novel recombinant CCV variants have been found and can cause lethal CCV infections in dogs [4]. Canine parvovirus type 2 (CPV-2) is a highly contagious and potentially fatal disease of dogs that has caused disease outbreaks accompanied by severe signs of gastroenteritis, especially in puppies [5]. Despite extensive vaccination, CPV-2 remains a leading infectious cause of canine mortality [6]. Consequently, early screening of CCV and CPV-2 is essential for preventing their transmission. At present, diagnosing CCV and CPV-2 infection by traditional methods (virus isolation, the colloidal gold method, etc.) has shown poor sensitivity, especially in the early stages of infection, and new diagnostic approaches based on PCR have been developed for sensitive detection in clinical samples [7, 8].

CCV and CPV-2 infections manifest as vomiting and acute diarrhea. CCV is typically associated with mild self-limiting diseases, while CPV-2 can lead to serious and often fatal diseases, and mixed infections are also common [9]. Early accurate diagnosis is very important for infected canines, but similar clinical symptoms increase the difficulty of diagnosis. Unlike methods for detecting human viral diseases, methods for detecting animal infectious diseases are imperfect [10]. To date, only a few multiplex PCRs have been used for the simultaneous detection of CCV and CPV-2. However, for most pet hospitals, PCR testing is difficult due to the lack of professional technicians and equipment. Fast, convenient, and fully automated detection methods are more suitable for screening animal diseases. Immunoassays can achieve A time-resolved fluorescence immunoassay (TRFIA) is a novel detection technique with high sensitivity and convenient and full automation that is highly suitable for detecting animal diseases [11, 12].

TRFIA is an immunological method that can be used to detect various diseases and biomarkers. For the diagnosis of animal infectious diseases using the TRFIA method, our team has established multiple TRFIA methods and kits, including single-label CCV antigen detection [13], single-label CPV-2 antigen detection [14], and African swine fever virus detection [15]. As the clinical symptoms of CCV and CPV-2 diseases are similar, single-label testing requires much time and expensive reagents; thus, it is necessary to establish a method that can simultaneously detect CCV and CPV-2. Therefore, in the present study, we established a double-labeling TRFIA for the simultaneous detection of CCV and CPV-2, and we verified that the detection performance of the dual-labeling method is equivalent to that of the single-labeling method.

## Materials and methods

### Antigens, antibodies, buffers and samples

The CCV antigen (nucleoprotein, N protein, expressed in *E. coli*) and CPV-2 antigen (expressed in VP2 protein, expressed in *E. coli*) and their paired antibodies were prepared by our team. Eu^3+^ and Sm^3+^ labeling reagents were obtained from PerkinElmer (Norwalk, USA). The reagents and buffer used in the study were prepared in the laboratory. Sixty-two fecal samples were obtained from suspected CCV-infected dogs, 75 fecal samples were obtained from suspected CPV-2-infected dogs, and 150 healthy fecal samples were obtained from Guangzhou Fu Mao Pet Hospital (Guangzhou, China).

### Coating and labeling procedure

CCV (No. 8D6 and 7E10) and CPV-2 (No. P2A3 and P3F6) paired antibodies were used for coating and labeling, respectively. Briefly, 8D6 and P2A3 antibodies were added to the 96-well microplate at a certain concentration and proportion, and the cells were incubated for 2 h at 37 °C. The microplate was blocked with blocking buffer (phosphate buffered saline (PBS) supplemented with 5% bovine serum albumin (BSA)) for 1 h at 37 °C. After drying, the microplate was stored under vacuum at 4 °C. Briefly, Eu^3+^ and Sm^3+^ labels were used for 7E10 and P3F6 antibody labeling, respectively. Eu^3+^/Sm^3+^ labeling reagent was added to the 7E10 and P3F6 antibodies, which were gently shaken at 4 °C and subsequently purified to obtain the Eu^3+^-labeled 7E10 antibodies and Sm^3+^-labeled P3F6 antibodies.

### Optimization of detection conditions

The reaction conditions were optimized according to a one-step procedure. The conditions that needed to be optimized included the concentration and proportion of 8D6 and P2A3 antibodies, the amount and proportion of labeling antibody to Eu^3+^/Sm^3+^ labels in the labeling procedure, the amount of Eu^3+^-labeled 7E10 antibodies and Sm^3+^-labeled P3F6 antibodies, the immunoreaction temperature and time, the washing time and the enhancement solution volume.

The optimized conditions were as follows: 8D6 antibody coating amount: 1 µg/mL, 100 µL/well; P2A3 antibody coating amount: 1.5 µg/mL, 100 µL/well; ratio of Eu^3+^ labeling reagent to 7E10 antibodies: 500 µg to 1.5 mg; ratio of Sm^3+^ labeling reagent to P3F6 antibodies: 500 µg to 2 mg; amount of Eu^3+^ labeling 7E10 antibodies: 90 µL; amount of Sm^3+^ labeling P3F6 antibodies: 110 µL; 6 washes; immunoreaction time: 50 min; and corresponding immunoreaction temperature: 37 °C.

### Kit preparation

The TRFIA kit included the following components: a coated 96-well microplate (vacuum sealing), Eu^3+^-labeled 7E10 antibodies, Sm^3+^-labeled P3F6 antibodies, sample diluent buffer (PBS), a series of standards (0, 0.1, 1, 10, 100, 200 and 500 ng/mL), washing buffer, enhancement solution and instructions.

### Test procedure

**Fig. 1:**
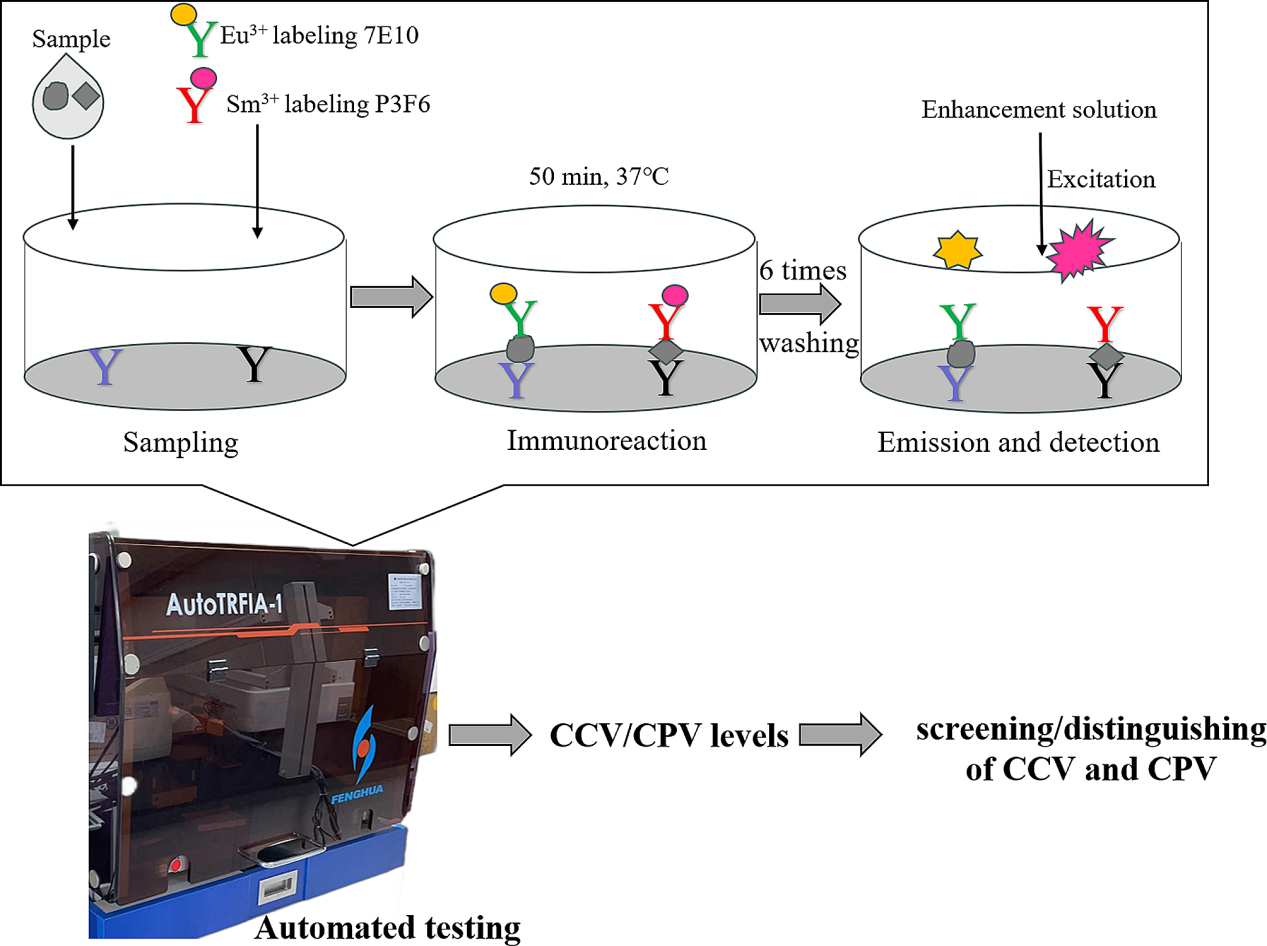
Schematic diagram of the TRFIA detection principle and steps

The test procedure was performed on a time-resolved analyzer, and the parameters were set in advance. The test procedure was as follows: 50 µL of sample, 90 µL/well of Eu^3+^-labeled 7E10 antibodies and 110 µL/well of Sm^3+^-labeled P3F6 antibodies were added to the coated 96-well plates and then incubated for 50 min at 37 °C. After 5 washes, 200 µL/well of enhancement solution was added to the wells, after which a time-resolved analyzer automatically measured the fluorescence values and calculated the CCV/CPV-2 concentration using built-in standard curves. A schematic diagram of the TRFIA detection principle and steps are shown in Fig. 1.

### Laboratory sensitivity assay

The CCV/CPV-2 antigen was diluted to concentrations of 0, 0.2, 2, 20, 200, 400 and 1000 ng/mL, and then, an equal volume of antigen was mixed to prepare a series of standards (0, 0.1, 1, 10, 100, 200 and 500 ng/mL). A series of standards were detected from the samples using a TRFIA kit, after which the corresponding fluorescence values were obtained. Three replicates were performed for each concentration. Standard curves were plotted using linear regression and log-log regression. The 0 ng/mL standard was detected 20 times as the sample, and the mean and standard deviation (SD) obtained from the detection were used for determining sensitivity (“sensitivity = mean + 2 × SD”) [16].

### Determination of the laboratory reference interval

A total of 150 healthy fecal samples were used for reference interval determination. After dissolution, the fecal supernatant was added to the coated 96-well microplate, and the TRFIA was performed. The CCV/CPV-2 concentration values were tested for normality using SPSS 20.0, and cutoff values were obtained according to the following formula: cutoff = mean + 1.64 × SD [17]. The reference interval was determined through the cutoff value.

### Laboratory specificity assay

Several common infectious disease samples from dogs were used for specificity experiments, including the following positive (P) clinical samples: canine distemper virus (CDV, 10 samples), canine parainfluenza virus (CPIV, 15 samples), canine adenovirus type 1 (CAV-1, 8 samples), and rabies virus (6 samples); 150 negative (N, healthy) samples; and the specific recombinant antigens CDV, CPIV, CAV-1 and rabies virus. The above clinical samples were confirmed by PCR testing and clinical symptoms. The samples were diluted/dissolved in PBS, after which TRFIA was performed. According to the reference interval, we determined the negativity and positivity of the samples and obtained the specificity results.

### Laboratory accuracy assay

Three concentrations of CCV/CPV-2 antigens (10 ng/mL, 100 ng/mL and 500 ng/mL) were added to the healthy fecal samples and detected using a TRFIA kit. Four replicates were performed for each sample. The coefficient of variation (CV) and recovery were calculated for accuracy validation. CV (%) = SD/mean × 100%. Recovery (%) = (determined concentration-basal concentration)/spiked concentration × 100%. The basal concentrations of CCV and CPV-2 were 4.23 ng/mL and 5.12 ng/mL, respectively.

### Laboratory stability assay

The kits were stored in the dark for 20 days at 4 °C or for 7 days at 37 °C. The CCV/CPV-2 antigen (100 ng/mL) was utilized for daily testing, after which the fluorescence values were recorded, and the curves were plotted to evaluate the stability of the kits.

### Methodology comparison and clinical sensitivity/specificity evaluation

Sixty-two fecal samples from suspected CCV-infected dogs and 75 fecal samples from suspected CPV-2-infected dogs were detected using PCR and a TRFIA kit, respectively. The results of the PCR method served as the detection criteria. Clinical sensitivity = true positive/(true positive + false-negative) × 100%), clinical specificity = true negative/(true negative + false-positive) × 100%). Pearson’s chi-square test was used to analyze the correlations between the two methods. The null hypothesis is that the two methods exhibit differences.

### Statistical methods

The data were statistically analyzed using SPSS 20.0. Pearson correlation analysis was performed, and Bland‒Altman plots and figures were constructed and plotted using GraphPad Prism 5 (GraphPad Software, USA). All the results are presented as the means ± *SDs*.

## Results

### Laboratory sensitivity

**Fig. 2:**
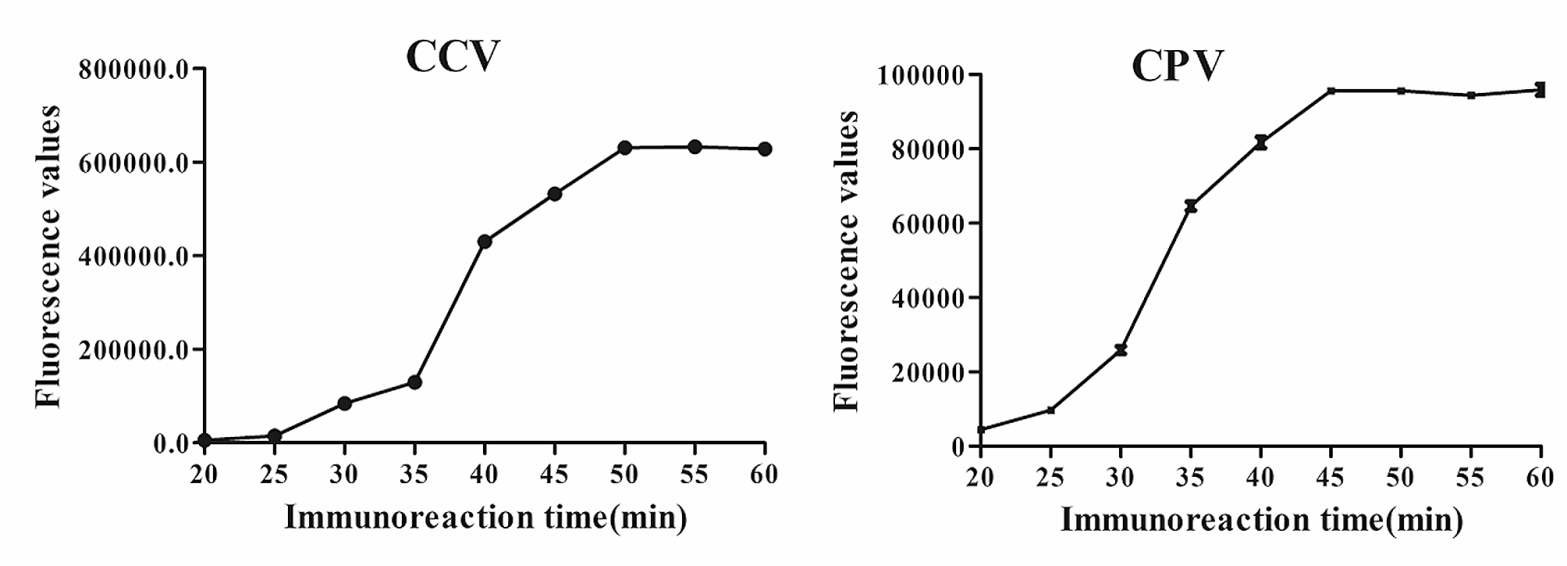
Optimization of the immunoreaction time

**Fig. 3:**
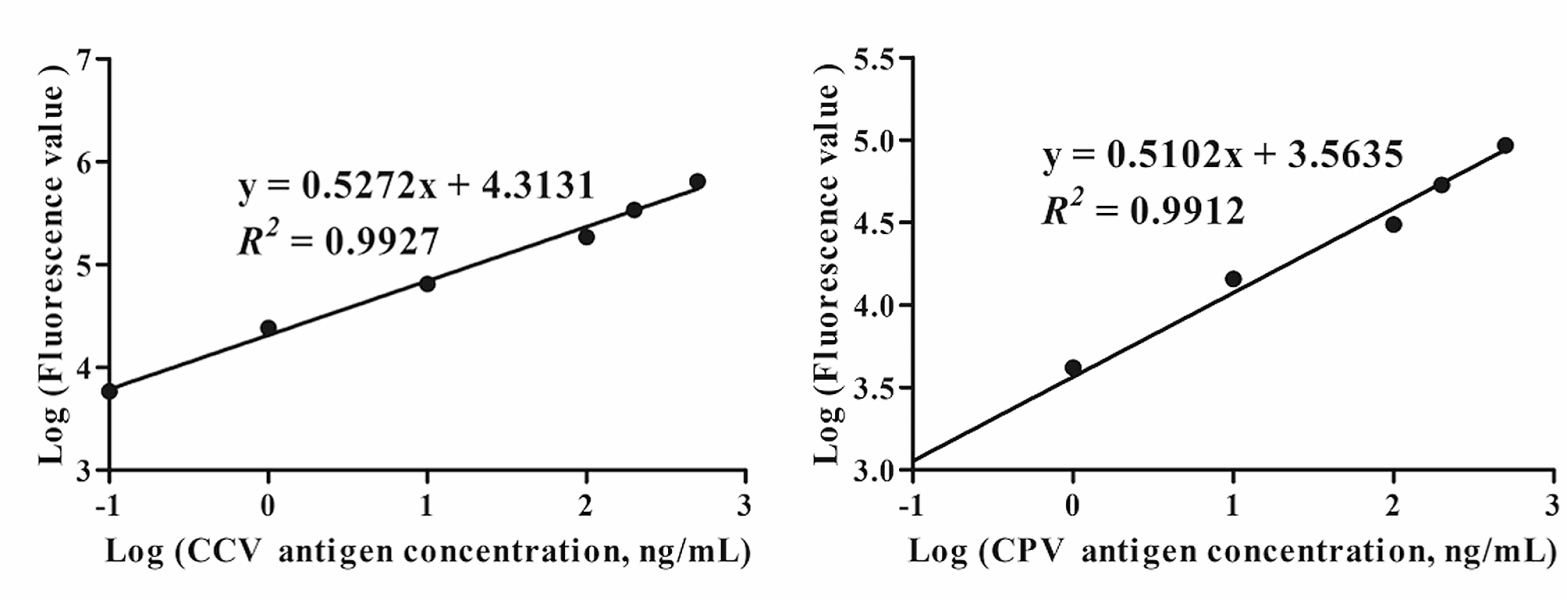
Standard curves for CCV and CPV-2 from this TRFIA

At the optimal immunoreaction time (50 min, Fig. 2), we plotted the standard curves as shown in Fig. 3. The standard curve equations used were y = 0.5272x + 4.3131 (*R*^*2*^ = 0.9927) and y = 0.5102x + 3.5635 (*R*^*2*^ = 0.9912). Based on the above standard curves, we calculated that the sensitivity of this TRFIA kit was 0.51 ng/mL for CCV and 0.80 ng/mL for CPV-2.

### Laboratory reference intervals

A normality test using SPSS 20.0 confirmed that 150 data points were normally distributed, and the cutoff value for CCV was 5.92 ng/mL (4.04 + 1.64 × 1.144), indicating that the positive reference interval for the CCV antigen of the TRFIA kit was > 5.92 ng/mL. The cutoff value for CPV-2 was 6.45 ng/mL (4.54 + 1.64 × 1.167), indicating that the positive reference interval for the CPV-2 antigen of the TRFIA kit was > 6.45 ng/mL.

### Laboratory specificity results

The specificity data are shown in Table 1. The common infectious disease samples and their specific recombinant antigens did not exhibit cross-reactivity, and the 50 negative samples did not lead to false-positives. Specifically, the TRFIA kit showed high specificity for detecting CCV and CPV-2.

**Table 1:** Specificity results

Interferents	No./Concentration	Determined indicator	P/N
CDV	10	CCV	N
		CPV-2	N
CPIV	15	CCV	N
		CPV-2	N
CAV-1	8	CCV	N
		CPV-2	N
Rabies virus	6	CCV	N
		CPV-2	N
Negative	150	CCV	N
		CPV-2	N
CDV antigen	100 ng/mL	CCV	N
		CPV-2	N
CPIV	100 ng/mL	CCV	N
		CPV-2	N
CAV-1	100 ng/mL	CCV	N
		CPV-2	N
Rabies virus	100 ng/mL	CCV	N
		CPV-2	N

### Laboratory accuracy results

As presented in Table 2, the *CV* ranged from 0.46 to 7.23%, and the recovery ranged from 101.21 to 110.28%. Table 2 indicates that the TRFIA kit has high accuracy.

**Table 2:** Accuracy results

	Spiked concentration	Mean ± SD	CV (%)	Recovery (%)
CCV	10	15.04 ± 1.09	7.23	108.06
(*n* = 4)	100	106.94 ± 1.65	1.54	102.71
	500	511.50 ± 4.69	0.92	101.45
CPV-2	10	15.26 ± 0.92	6.01	110.28
(*n* = 4)	100	108.49 ± 1.89	1.74	104.26
	500	510.26 ± 2.35	0.46	101.21

### Laboratory stability results

**Fig. 4:**
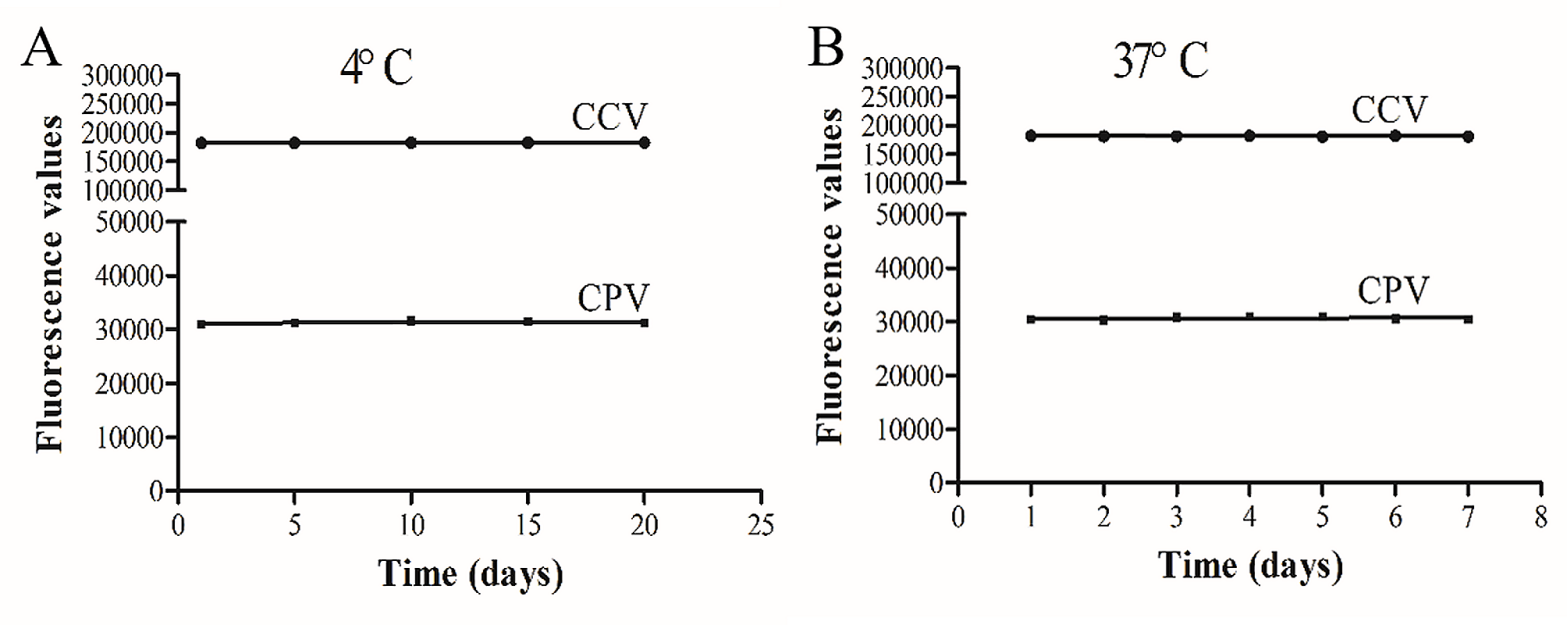
Stability of the kits (4 and 37 °C)

After 20 days of 4 °C storage, there was no significant change in the fluorescence values (Fig. 4A). After 7 days of storage at 37 °C, there was no significant change in the fluorescence values (Fig. 4B). These results indicated that the stability of the kits was good. Specifically, the kit can be temporarily stored for 20 days at 4 °C and can be stored for 12 months at temperatures less than − 20 °C.

### Methodology comparison and clinical sensitivity/specificity evaluation results

PCR results revealed 47 positive CCV samples, 15 negative CCV samples, 42 positive CPV-2 samples, and 33 negative CPV-2 samples. As presented in Table 3, Pearson’s chi-squared test showed that the Pearson χ2 value was 47.124 (*P* < 0.001) for CCV, and the Pearson χ2 value was 59.91 (*P* < 0.001) for CPV-2. At the degree of freedom 1, the χ2 value for CCV and CPV-2 were 47.1 and 59.91, respectively, which was higher than the critical value of 10.8 at *P* < 0.001, leading us to accept the alternative hypothesis that there was no statistically significant difference between the developed TRFIA method and PCR based detection. Additionally, for CCV detection, the clinical sensitivity of the TRFIA method was 95.74%, and the clinical specificity was 93.33%. For CPV-2 detection, the clinical sensitivity of the TRFIA method was 92.86%, and the clinical specificity was 96.97%.

**Table 3:** Pearson’s chi-square test was used for comparisons of the PCR method and the TRFIA kit

		PCR		Total	Pearson χ2
		Positive	Negative		
CCV-TRFIA	Positive	45	1	46	
	Negative	2	14	16	47.124 (*P* < 0.001)
	Total	47	15	62	
CPV-2-TRFIA	Positive	39	1	40	
	Negative	3	32	35	59.91 (*P* < 0.001)
	Total	42	33	75	

## Discussion

Similar to humans, animals are at risk of severe infectious diseases. Tests for animal diseases involve additional challenges, such as simplicity, long testing time and portability. In recent decades, dramatic improvements in animal disease detection have been achieved, but this improvement is insufficient because additional new potential pathogens will emerge in the future [18]. In 2022, Manessis G et al. proposed the concept of point-of-care testing (POCT) for animal diseases [10]. POCTs are analytical devices that provide rapid diagnostic capabilities without the need for professional technicians or core laboratory facilities [19, 20]. From these perspectives, POCT is suitable for animal disease detection in environments such as grassroots health care and pet clinics. Emerging human medicine technologies are expected to overcome some of the challenges associated with animal POCT. In this study, we established a fully automated TRFIA method for screening/distinguishing CCV/CPV-2, which may help improve strategies for preventing and controlling infectious animal diseases in the future.

As lanthanides have a long fluorescence lifetime, large Stokes shift and sharp emission profile, they are favorable for use in fluorescent labeling for microsecond bioassays because of their higher sensitivity [21, 22]. TRFIA is a new detection technology that utilizes lanthanide elements for fluorescent labeling. Most importantly, the excitation and emission wavelengths of different lanthanide elements are significantly different, allowing simultaneous detection of multiple indicators in one bioassay; Eu^3+^ and Sm^3+^ are the most commonly used lanthanide elements [23, 24].. The advantage of other single-labeling immunoassays is that they cannot match each other well, as indicated by their significant time and cost effectiveness. Additionally, the use of POCTs for treating animal diseases has become possible due to the emergence of portable fluorescent lanthanide readers [25]. We hope to develop a more convenient detector and increase the practicality of this TRFIA kit.

Currently, there is no double-label TRFIA method for detecting animal diseases. In this study, we optimized and established a double-label TRFIA for CCV and CPV-2 detection using Eu^3+^ and Sm^3+^ and assembled the TRFIA into a kit. The assembled TRFIA kit showed high sensitivity and high specificity; all *CVs* were less than 10%, and the recovery ranged from 101.21 to 110.28%. The kits can be temporarily stored for 20 days at 4 °C and can be stored for 12 months at temperatures less than − 20 °C. A methodology comparison of 137 clinically suspected patients revealed that there was no statistically significant difference between the TRFIA kit and the PCR method. Additionally, the clinical sensitivities were 95.74% and 92.86%, and the clinical specificities were 93.33% and 96.97%. These data showed that the clinical performance of the TRFIA kit is equivalent to that of the standardized PCR method. Considering the time and cost-effectiveness, compared with multiplex PCR and standardized PCR, this fully automated TRFIA method is more convenient and efficient (total test time 1 h vs. 2 h, full-automation vs. multiple steps, including nucleic acid extraction, centrifugation, and machine testing, etc., < 1 dollar/sample vs. several dollars/sample). An indirect enzyme-linked immunosorbent assay (ELISA) for canine coronavirus antibody detection established by Hao et al., the positive rate was 82.8% [26]. Shima et al. prepared the immunochromatographic kit for CPV detection, the clinical sensitivity was 95.4%, and the specificity was 71.4% [27]. Another immunochromatographic method for CPV detection, the sensitivity was < 80.4% [28]. Comparative analysis has shown that this fully automated TRFIA method exhibited superior detection performance. Consequently, this study provides a sensitive and accurate new method for screening/distinguishing CCV from CPV-2.

In conclusion, a fully automated TRFIA method was established for screening/distinguishing CCV/CPV-2 with high sensitivity, specificity, accuracy and stability. The clinical sensitivity/specificity reached more than 90%, indicating the potential for clinical application. This kit provides a new option for screening/distinguishing between CCV and CPV-2 and may help improve strategies to prevent and control infectious animal diseases in the future.

## Data Availability

All data generated or analysed during this study have been provided in the text.

## References

[1] Decaro N, Buonavoglia C (2011). Canine coronavirus: not only an enteric pathogen. Vet Clin North Am Small Anim Pract.

[2] Haake C, Cook S, Pusterla N, Murphy B (2020). Coronavirus infections in Companion animals: Virology, Epidemiology, Clinical and pathologic features. Viruses.

[3] Decaro N, Lorusso A (2020). Novel human coronavirus (SARS-CoV-2): a lesson from animal coronaviruses. Vet Microbiol.

[4] Licitra BN, Duhamel GE, Whittaker GR (2014). Canine enteric coronaviruses: emerging viral pathogens with distinct recombinant spike proteins. Viruses.

[5] Miranda C, Parrish CR, Thompson G (2016). Epidemiological evolution of canine parvovirus in the Portuguese domestic dog population. Vet Microbiol.

[6] Decaro N, Buonavoglia C, Barrs VR (2020). Canine parvovirus vaccination and immunisation failures: are we far from disease eradication?. Vet Microbiol.

[7] Molesan A, Goodman L, Ford J, Lovering SJ, Kelly K (2019). The causes of Canine myocarditis and Myocardial Fibrosis are elusive by targeted Molecular Testing: Retrospective Analysis and Literature Review. Vet Pathol.

[8] Decaro N, Buonavoglia C (2012). Canine parvovirus–a review of epidemiological and diagnostic aspects, with emphasis on type 2c. Vet Microbiol.

[9] Cavalli A, Desario C, Kusi I (2014). Detection and genetic characterization of canine parvovirus and canine coronavirus strains circulating in district of Tirana in Albania. J Vet Diagn Invest.

[10] Manessis G, Gelasakis AI, Bossis I (2022). Point-of-Care Diagnostics for Farm Animal diseases: from biosensors to Integrated Lab-on-chip devices. Biosens (Basel).

[11] Fu L, Qian Y, Zhou J, Zheng L, Wang Y (2020). Fluorescence-based quantitative platform for ultrasensitive food allergen detection: from immunoassays to DNA sensors. Compr Rev Food Sci Food Saf.

[12] Huertas-López A, Martínez-Carrasco C, Cerón JJ, Sánchez-Sánchez R, Vázquez-Calvo Á, Álvarez-García G, Martínez-Subiela S (2019). A time-resolved fluorescence immunoassay for the detection of anti-neospora caninum antibodies in sheep. Vet Parasitol.

[13] Chen CC, Liang Hk, Gao HW, Lai HR, Zhong SH, LI JX, Li LQ (2023). Development and preliminary application of time-resolved immunofluorescence analysis for canine coronavirus. Chin J Anim Infect Dis.

[14] Chen C, Liang H, Hu B, Ning B, Lai H, He Y, Guo G, Zhong S, Li L (2021). Determination of parvovirus antigen in the vaccine using time-resolved fluorescence immunoassay. Biotechnol Appl Biochem.

[15] Chen C, Lai H, Liang H, He Y, Guo G, Li L (2021). A New Method for Detection African Swine Fever Virus: Time-resolved fluorescence immunoassay. J Fluoresc.

[16] Li HT, Huang ZF, Lin BC, Chen XY, Xiong XY, Cao AF, Yang CZ (2020). Simultaneous detection of fungal (1,3)-β-D-glucan and procalcitonin using a dual-label time-resolved fluorescence immunoassay. Biotech Appl Biochem.

[17] Ma HY, Mao Q, Zhu YB, Cong CL, Zheng SY, Zhang Q, Chen CC, Li LQ (2021). Time-resolved fluorescence immunoassay (TRFIA) for the simultaneous detection of MMP-9 and Lp-PLA2 in serum. J Fluoresc.

[18] Perry BD, Grace D, Sones K (2013). Current drivers and future directions of global livestock disease dynamics. Proc Natl Acad Sci U S A.

[19] Ferreira CES, Guerra JCC, Slhessarenko N, Scartezini M, Franca CN, Colombini MP, Berlitz F, Machado AMO, Campana GA, Faulhaber ACL, Galoro CA, Dias CM, Shcolnik W, Martino MDV, Cesar KR, Sumita NM, Mendes ME, Faulhaber MHW, Pinho JRR, Barbosa IV, Batista MC, Khawali C, Pariz VM, Andriolo A (2018). Point-of-care testing: General aspects. Clin Lab.

[20] Goble JA, Rocafort PT (2017). Point-of-care testing. J Pharm Pract.

[21] Yuan J, Wang G (2005). Lanthanide complex-based fluorescence label for time-resolved fluorescence bioassay. J Fluoresc.

[22] Ge X, Wei R, Sun L (2020). Lanthanide nanoparticles with efficient near-infrared-II emission for biological applications. J Mater Chem B.

[23] Fan J, Xiao H, Zhang J, Zhou B, Deng L, Zhang Y, Huang B (2017). A magnetic nanoparticle-labeled immunoassay with europium and samarium for simultaneous quantification of serum pepsinogen I and II. Br J Biomed Sci.

[24] Yang X, Ye Y, Wang T, Li M, Yu L, Xia M, Qian J, Hu Z (2019). Eu3+ /Sm3 + dual-label time-resolved fluoroimmunoassay for measurement of hepatitis C virus antibodies. J Clin Lab Anal.

[25] Li X, Chen X, Wu J, Liu Z, Wang J, Song C, Zhao S, Lei H, Sun Y (2021). Portable, Rapid, and sensitive time-resolved fluorescence immunochromatography for On-Site detection of dexamethasone in milk and pork. Foods.

[26] Hao YF, Li SH, Zhang GZ, Xu Y, Long GZ, Lu XX, Cui SJ, Qin T (2021). Establishment of an indirect ELISA-based method involving the use of a multiepitope recombinant S protein to detect antibodies against canine coronavirus. Arch Virol.

[27] Shima FK, Gberindyer FA, Tion MT, Fagbohun OA, Omobowale TO, Nottidge HO (2021). Diagnostic performance of a Rapid Immunochromatographic Test Kit for detecting Canine Parvovirus infection. Top Companion Anim Med.

[28] Decaro N, Desario C, Beall MJ, Cavalli A, Campolo M, Dimarco AA, Amorisco F, Colaianni ML, Buonavoglia C (2010). Detection of canine parvovirus type 2c by a commercially available in-house rapid test. Vet J.

